# The British Hospitals Association

**Published:** 1910-10-08

**Authors:** 


### SPECIAL INSTITUTIONAL ARTICLES.

### THE BRITISH HOSPITALS ASSOCIATION.

### FIRST ANNUAL CONFERENCE IN GLASGOW.

THE first annual Conference in connection with the British Hospitals Association was commenced in the Humanity Class Room of the University Buildings, Glasgow, on Thursday, the 29th ult. In the absence of the President of the Association, Mr. H. Cosmo Bonsor, who was unfortunately prevented from attending, Mr. J. D. Hedderwick, a member of the Council, presided. In his opening remarks the Chairman said that the Association, although a young one, already occupied the place it had aimed at. It was thoroughly representative of the hospitals throughout the country, and he hoped that very soon every hospital in the United Kingdom would have its worthy representative on the roll of members. They all felt that there were special benefits to be obtained by meeting in conference and exchanging experience. It was for that purpose that they had met that day, and he would only add that the Glasgow members of the Association, who appreciated the honour of the first annual conference of the Association being held in the city of Glasgow, most heartily welcomed their fellow-members from all part of the country.

Dr. Mackintosh, M.V.O., Hon. Secretary, read letters of regret and apology from the President (Mr. Bonsor), Sir Henry Burdett, and other members who had been unable to attend.

### THE LORD PROVOST'S WELCOME.

The Lord Provost: Mr. Chairman, Ladies and Gentlemen,—Connected as I am in my official capacity with most of the large infirmaries and hospitals in Glasgow, it is perhaps not inappropriate that I should at the outset of your proceedings extend, on behalf of the citizens of Glasgow, a cordial welcome to the members of the British Hospitals Association on the occasion of its first meeting here. In a city like Glasgow, which saw the birth of the great antiseptic method of surgery which is associated with the distinguished name of Lord Lister and other surgeons and physicians, it would be strange indeed if your Association were not welcome here, especially meeting, as you do, within the precincts of the University that has sheltered and trained, both as teachers and students, men whose names are synonymous all the world over with the highest exemplification of medical and surgical science. (Applause.) We believe we can show the members of the Association something not altogether obsolete in hospital construction and management. Our Royal Infirmary, the oldest in the city, with its honourable record of noble and magnificent service, is especially worthy of attention, particularly the portion already reconstructed. When completed it will take rank amongst the foremost institutions of its kind in the United Kingdom. The Western and Victoria Infirmaries are not less embodiments of all the most modern and up-to-date requirements of science. It says much for the liberality of our citizens that such magnificent accommodation is provided for the sick and suffering, and it speaks volumes for the humane and sympathetic instincts of our medical men that they are prepared to give their best skill and service to a cause so worthy. Our Corporation and Parish Council hospitals and many similar institutions, dedicated to special forms of disease, should also not escape the attention of the Association, and I am sure that I speak for the managers of these hospitals when I say that they will be happy to afford you every facility for the inspection of their institutions. I shall have another opportunity in the City Chambers of speaking on this subject, and I will therefore content myself once more with extending to all the members a most cordial welcome to the City of Glasgow. I trust that your deliberations will be most successful here. (Applause.)

The Chairman: Ladies and Gentlemen,—The Lord Provost of Glasgow is a very busy man, and I am sorry that he will have to betake himself to another sphere of usefulness very soon; but before he goes I wish to ask you to recognise in his person the graceful welcome which the City of Glasgow is extending to this Association to-day, by asking him to accept of honorary membership of the Association. (Applause.) We have power to elect honorary members. We have not exercised that power yet—in fact, we have not had time, as this is the first opportunity we have had of exercising that power, and I do not think we could exercise it upon a better man, and in a better way, than by asking the Lord Provost to accept honorary membership. (Applause.)

The Lord Provost: Mr. Chairman, Ladies and Gentlemen,—I desire to thank you for the great honour that you have not only done me personally, but done the City of Glasgow, in conferring honorary membership of the Association on me, as Lord Provost. I take it as a very great compliment personally, and I am quite satisfied that the citizens of Glasgow will do likewise. (Applause.)

### SOCIAL POLICY OF HOSPITALS.

The first paper read was by Mr. C. S. Loch, LL.D., D.C.L., on "The Social Policy of Voluntary Hospitals." Therein were discussed the questions of the relation of the hospital to the medical profession and medical education, to the Poor Law and medical charities, and to the individual. The paper submitted the outlines of a social policy which the author trusts will lead to a better remuneration of medical men, to a better division of labour among institutions for the sick, and to a serviceable association in charity. It was well discussed by the Chairman, Mr. James Cunningham, Mr. C. Thies, Professor James, and others.*

Mr. James Cunningham, a member of the Glasgow Parish Council, said that there was a consensus of opinion that medical men acting under the Poor Law authority were not adequately remunerated for the work they had to do. Many people were using the voluntary hospitals who were in a position to pay something towards the expenses of medical treatment. He supported Mr. Loch's suggestion for a scheme of hospital almoners. Professor Henry Jones said the main question apparently was in how far it would be possible to extend the infirmary system so as to enable all citizens to make use of the infirmaries if they desired, and also to pay for the treatment they received in these institutions. It was absurd to arrest the development of hospital aid on the ground that it would throw medical men out of work. He frankly denied the presupposition that State-aid implied impoverishment of character in all respects. Mr. Thies strongly supported the method at present in use in Germany and Switzerland. The establishment of the King's Fund, he thought, had only staved off the matter, not solved the problem. Out of 65,000 hospital beds in London 55,000 were already being paid for out of the rates. He briefly described the German method, and pointed out that at present there was hardly a

* A full report of this and other papers and the accompanying discussions will appear in a future issue.

voluntary hospital in London which had not a serious financial difficulty to face. Mr. Loch briefly replied to the discussion, and stated that he had merely attempted to outline a workable policy which would be safe and acceptable.

### Hospital Abuse.

Mr. Scott Finnie, of Aberdeen, then read a paper on "The Abuse of the Hospital and its Cure." Taking five of the great Scottish hospitals, he showed how the number of out-patients had increased during the last decade out of all proportion to the increase in the number of in-patients. The advance in the out-patient departments he considered a proof of the increase of hospital abuse, or at least suspiciously suggestive of such abuse. At present the hospitals were almost powerless to cope with such abuse, and he looked forward to the time when concerted action would be taken by the hospital authorities to cope with the matter. It should be possible to initiate a system under which all legitimate interests would be adequately provided for and under which an end would be put to flagrant abuse of charity.

This paper evoked a keen and instructive discussion, in which all aspects of the question of institutional abuse were considered by the various speakers. Dr. Dewar spoke at some length, and outlined the scheme which had been attempted at Edinburgh to deal with the problem by establishing a hospital for paying patients. Mr. J. A. Roxburgh, however, refused to admit that there was much abuse at present, at least in Glasgow, and pointed out that the proposal to establish paying wards at the Glasgow Western Infirmary had been abandoned, after full discussion, since it had been found unworkable. Mr. W. B. Blaikie, of the Edinburgh Royal Infirmary, expressed the opinion that the only radical way of dealing with the problem was by establishing paying wards and adopting the German system. Mr. Kershaw, of the London Throat Hospital, thought that ignorance rather than wilful design was responsible for the increase in abuse. In his opinion, the whole system of hospital relief was drifting towards State control. The discussion was finally adjourned until the following day. On the invitation of the Chairman of the Western Infirmary, Sir Mathew Arthur, and the Chairman of the Royal Infirmary, Mr. Hedderwick, the members of the Association were entertained to luncheon at the Students' Union in the University grounds. After this function the members visited the Western Infirmary, and later on parties, at the invitation of the Committee of the Royal Asylum, motored to the new sewage works on the Clyde to inspect the recent additions to the plant.

### FRIDAY'S SESSION.

The concluding session of the Conference, held on Friday, was under the presidency, at first of Mr. J. A. Roxburgh, and later on of Mr. Hedderwick. The first paper read was by Mrs. Sidney Webb on "The Unified Medical Service and how it will affect the Hospitals." This dealt with the lines of policy laid down in the Minority Report of the Royal Commission on the Poor Law, and pleaded for the institution of a unified county medical service, which, the writer thought, would not be detrimental to the interests of the voluntary hospital. A very interesting discussion followed the reading of this paper, members at the same time dealing with Mr. Stewart's paper on the Minority Report, and with that of Mr. Finnie on "Hospital Abuse," which had been read on the preceding day. Mr. J. R. Motion, clerk to the Glasgow Parish Council, criticised Mrs. Webb's premises at length, and thought that the statements made were very largely exaggerations calculated to attract public attention. He strongly disapproved of the outlined policy, and condemned the recommendations of the Minority Report, which, he hoped, would never be adopted in Scotland, where local administration had all along been ahead of England. Councillor Macpherson, of Edinburgh, Dr. Robertson, Medical Officer of Health, Leith, Dr. Nathan Raw, Professor Glaister, Councillor Anderson, of Glasgow, and the Hon. Secretary joined in the discussion, the majority strongly upholding the principle of the voluntary system.

### The Question of Consumption.

The second paper was by Dr. Nathan Raw on "The Treatment of Tuberculosis." The author emphasised the fact that consumption was largely a matter of environment and infection, and stated that it was of vital importance that consumptive children should be treated in special schools. In the discussion that followed many members took part, and generally agreed with the conclusions summarised by the author. Mr. McArly pointed out the urgent want of beds in consumption sanatoria, while Dr. Duncan thought that the principle of open-air treatment might easily be extended to the voluntary hospitals of Glasgow. He strongly favoured isolation, and pointed out that a large number of advanced cases of the disease were now neglected, and were the means of spreading phthisis in slum districts.

### Hospital Ambulances.

Following this came a very interesting debate on the subject of hospital ambulances. Mr. Nicol Brown, Chairman of the Council of St. Andrews Ambulance Association, gave his experience of the motor and horse-drawn ambulance: the cost of the former worked out at a shilling and a halfpenny per mile, against a shilling and fivepence per mile for the motor ambulance, these figures including all charges except depreciation. Sir George Beatson favoured the establishment of a voluntary ambulance system for all large cities, and thought the horse-drawn vehicles were more reliable than the motor ambulances, although the latter were preferable where speed was demanded. Dr. Goodall and other speakers favoured the motor ambulance, which they held to be much superior in many respects to the older type of horse-drawn ambulance. The discussion elicited much interesting information on technical points. A further discussion, initiated by Dr. Ebenezer Duncan, then took place on the question of paying patients in fever hospitals. Dr. Duncan suggested that paying patients should be admitted to all hospitals, and should be allowed to have their own medical attendants.

Formal Business.

At the conclusion of the discussions on the papers read, the Chairman proposed a hearty vote of thanks to the University authorities for their kindness and courtesy in allowing the Association the use of their buildings and extending hospitality to the Conference. The vote was passed with acclamation, and Principal Sir Donald Macalister, in response, apologised for his unavoidable absence at the opening of the Conference, and expressed the pleasure he felt in welcoming the Association to Glasgow. The following office-bearers were elected for the ensuing year: President, Mr. H. Cosmo Bonsor, Chairman, Guy's Hospital; Hon. Treasurer, Mr. C. W. Thies, Royal Free Hospital; Hon. Secretaries, Mr. A. W. West and Dr. D. J. Mackintosh, M.V.O. Formal votes of thanks closed the proceedings.

The Corporation Luncheon.

At the conclusion of the morning's business a luncheon was given in the City Chambers to the members and delegates by the Glasgow Corporation. The Lord Provost presided, and among those present were Lord Inverclyde, Mr. J. D. Hedderwick, and nearly all the members of the Association, Sir Donald Macalister, Sir John Primrose, Professor Noel Paton, Sir Mathew Arthur, the Right Hon. Chas. Scott Dickson, Sir William Bilsland, Lady Chisholm, and many others. The Lord Provost, in proposing the toast of "The British Hospitals' Association," said it was a healthy sign of the times to find experts on hospital work meeting in conference to exchange views, and dealing with important and pressing problems of national interest. He felt that the Association had a splendid scope, and could do much useful work, and he was convinced that it would in course of time come to have a very beneficial influence on medical charities all over the country. Messrs. West and Hedderwick suitably responded. The toasts of "The Corporation" and "The Lord Provost" were proposed by Mr. Thies and Mr. Scott Dickson respectively, and acknowledged by the Lord Provost, whereupon the proceedings terminated.

During the intervals between the sessions the members of the Association visited the most important charitable institutions in the city.

